# Chronic alcohol use primes bronchial cells for altered inflammatory response and barrier dysfunction during SARS-CoV-2 infection

**DOI:** 10.1152/ajplung.00381.2022

**Published:** 2023-10-03

**Authors:** Kristen F. Easley, R. Clayton Edenfield, Megan E. J. Lott, Ryan C. Reed, Jayasri Das Sarma, Ashish J. Mehta, Bashar S. Staitieh, Erin K. Lipp, In Ki Cho, Scott K. Johnson, Cheryl A. Jones, Anne-Gaelle Bebin-Blackwell, Joshua M. Levy, S. Mark Tompkins, Charles A. Easley, Michael Koval

**Affiliations:** ^1^Division of Pulmonary, Allergy, Critical Care and Sleep Medicine, Emory University School of Medicine, Atlanta, Georgia, United States; ^2^Department of Environmental Health Science, College of Public Health, University of Georgia, Athens, Georgia, United States; ^3^Regenerative Bioscience Center, University of Georgia, Athens, Georgia, United States; ^4^Department of Biological Sciences, Indian Institute of Science Education and Research Kolkata, Mohanpur, West Bengal, India; ^5^Department of Ophthalmology, University of Pennsylvania, Philadelphia, Pennsylvania, United States; ^6^Atlanta Veterans Affairs Health Care System, Decatur, Georgia, United States; ^7^Center for Vaccines and Immunology, University of Georgia, Athens, Georgia, United States; ^8^Department of Otolaryngology, Emory University School of Medicine, Atlanta, Georgia, United States; ^9^National Institute on Deafness and Other Communication Disorders, National Institutes of Health, Bethesda, Maryland, United States; ^10^Department of Cell Biology, Emory University School of Medicine, Atlanta, Georgia, United States

**Keywords:** airway epithelia, alcohol use disorder, COVID-19, inflammation, tight junction

## Abstract

Alcohol use disorder (AUD) is a significant public health concern and people with AUD are more likely to develop severe acute respiratory distress syndrome (ARDS) in response to respiratory infections. To examine whether AUD was a risk factor for more severe outcome in response to severe acute respiratory syndrome coronavirus 2 (SARS-CoV-2) infection, we examined early responses to infection using cultured differentiated bronchial epithelial cells derived from brushings obtained from people with AUD or without AUD. RNA-seq analysis of uninfected cells determined that AUD cells were enriched for expression of epidermal genes as compared with non-AUD cells. Bronchial epithelial cells from patients with AUD showed a significant decrease in barrier function 72 h postinfection, as determined by transepithelial electrical resistance. In contrast, barrier function of non-AUD cells was enhanced 72 h after SARS-CoV-2 infection. AUD cells showed claudin-7 that did not colocalize with zonula occludens-1 (ZO-1), indicative of disorganized tight junctions. However, both AUD and non-AUD cells showed decreased β-catenin expression following SARS-CoV-2 infection. To determine the impact of AUD on the inflammatory response to SARS-CoV-2 infection, cytokine secretion was measured by multiplex analysis. SARS-CoV-2-infected AUD bronchial cells had enhanced secretion of multiple proinflammatory cytokines including TNFα, IL-1β, and IFNγ as opposed to non-AUD cells. In contrast, secretion of the barrier-protective cytokines epidermal growth factor (EGF) and granulocyte macrophage-colony stimulating factor (GM-CSF) was enhanced for non-AUD bronchial cells. Taken together, these data support the hypothesis that AUD is a risk factor for COVID-19, where alcohol primes airway epithelial cells for increased inflammation and increased barrier dysfunction and increased inflammation in response to infection by SARS-CoV-2.

**NEW & NOTEWORTHY** Alcohol use disorder (AUD) is a significant risk factor for severe acute respiratory distress syndrome. We found that AUD causes a phenotypic shift in gene expression in human bronchial epithelial cells, enhancing expression of epidermal genes. AUD cells infected with severe acute respiratory syndrome coronavirus 2 (SARS-CoV-2) had higher levels of proinflammatory cytokine secretion and barrier dysfunction not present in infected non-AUD cells, consistent with increased early COVID-19 severity due to AUD.

## Graphical abstract

**Figure:**
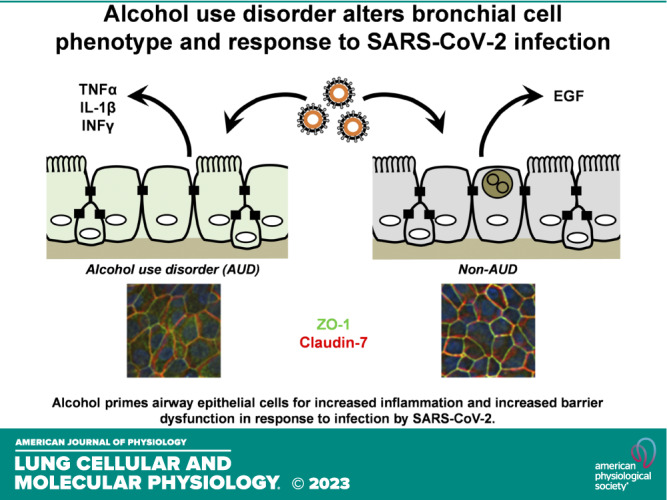

## INTRODUCTION

COVID-19 is caused by severe acute respiratory syndrome coronavirus 2 (SARS-CoV-2) and is associated with respiratory failure in the most severe cases (1, 2). It is estimated that a third of patients with COVID-19 requiring mechanical ventilation do not survive (3). Several comorbidities are associated with increased COVID-19 severity, including obesity, diabetes, and other chronic diseases (4). It has long been appreciated that alcohol use disorder (AUD) is a risk factor for increased severity of lung disease (5–7). This includes increased susceptibility to infectious pneumonia (8–11) and poor outcomes in acute respiratory distress syndrome (ARDS) (12). As ARDS is a significant pathological consequence of SARS-CoV-2 infection (13), it is noteworthy that people with AUD and COVID-19 have been shown to have a higher rate of hospitalization and mortality (14), suggesting that AUD is a risk factor for increased severity of COVID-19 related illness (15). Compounding the potential impact of AUD on patient outcomes is the added stress of experiencing the COVID-19 pandemic, which has been associated with substance abuse, including increased alcohol consumption (16, 17). However, the mechanisms by which AUD influences lung epithelial responses to SARS-CoV-2 are not known at present.

Numerous factors associated with AUD contribute to increased inflammation and overall poor lung health. Patients with AUD and animal models of AUD show higher levels of inflammatory cytokines and chemokines in the airway (18–21), alveolar macrophage dysfunction (22, 23), and disruption of the lung microbiome (24). In addition, chronic alcohol exposure results in lung epithelial barrier dysfunction, which predisposes patients with ARDS to more severe pulmonary edema (25, 26). For instance, lung epithelial barrier permeability is regulated by the apical junctional complex, composed of tight junctions and adherens junctions (27–29). The impact of alcohol exposure on the expression, organization, and function of lung epithelial tight junction proteins can be demonstrated using in vitro models (21, 30–32). Of note, alveolar epithelial cells isolated from alcohol-fed animals have barrier dysfunction that persists in tissue culture, even in the absence of added ethanol (33).

It is clear that SARS-CoV-2 has the capacity to infect multiple epithelia throughout the respiratory tree (34). However, whether the impact of AUD on SARS-CoV-2 infection is due to the effects of alcohol on lung epithelia has not been determined. Since AUD is a significant risk factor for ARDS in general (35) and those with AUD had higher rates of hospitalization following infection with SARS-CoV-2 (14), we hypothesize that AUD sensitizes lung epithelial cells to the effects of SARS-CoV-2 infection.

It is well established that cultured human lung epithelial cells have significant utility for the study of the effects of SARS-CoV-2 infection (36–43), suggesting that an in vitro model would shed light on the impact of AUD on the effects of SARS-CoV-2 infection on lung epithelial cells. To do this, we isolated bronchial brushings from patients with and without AUD, expanded them in a basal cell state and then differentiated them with an air liquid interface (ALI) culture system using methods previously established by our laboratory (44). These samples were subject to RNA-seq analysis to determine baseline transcriptome differences. The cells were infected with SARS-CoV-2 and the effect on barrier function, junction protein expression, and inflammatory cytokine production were measured. We found that after infection, cells from patients with AUD had a significant decrease in transepithelial resistance (TER) and showed enhanced secretion of several proinflammatory cytokines including TNFα, IL-1β, and IFNγ as compared with non-AUD cells. In contrast, infected non-AUD cells produced significantly higher levels of granulocyte macrophage-colony stimulating factor (GM-CSF) and epidermal growth factor (EGF), two anti-inflammatory, barrier-protective cytokines, than AUD cells. Taken together, these data support the hypothesis that AUD is a risk factor for COVID-19 and that this is, in part, due to an effect of chronic alcohol exposure on airway epithelial cells.

## MATERIALS AND METHODS

### Donor Consent

Research involving human research participants was performed in accordance with the Declaration of Helsinki guidelines. Written informed consent was obtained from participants. All human subject protocols were reviewed and approved by the Emory University Institutional Review Board and the Atlanta Veterans Affairs Health Care System Research and Development Committee. Potential subjects for study enrollment were screened using the Short Michigan Alcohol Screening Test and AUD Identification Test (45, 46). Individuals with a history of AUD were recruited from the Substance Abuse Treatment Program at the Atlanta Veterans Affairs Health Care System, and otherwise healthy control subjects were recruited from general Veterans Affairs medical clinics (47). Additional subject inclusion criteria included active alcohol consumption, in which the last alcoholic drink was <8 days before bronchoscopy. Subjects were excluded if they primarily consumed intoxicating substances other than alcohol, were HIV positive, or had abnormal chest radiographs.

### Airway Epithelial Cell Culture and Infection

Cells from bronchial brushings were expanded in coculture with irradiated 3T3 fibroblast feeder cells in F + Y reprogramming media (FYRM) as previously described (44). FYRM was changed every other day until the cells were ∼70%–90% confluent and then the cells were isolated by first removing the 3T3 feeder layer using calcium/magnesium-free phosphate buffered saline supplemented with 1 mM EDTA (PBS/EDTA), followed by detaching epithelial cells by incubating with Accutase (Sigma-Aldrich No. A6964) at room temperature (RT) for 10 min. Cells were then centrifuged and frozen as P1 stocks. For experiments, cells were thawed, expanded using FYRM, and then seeded on Transwell permeable supports precoated with type IV collagen (Sigma-Aldrich No. C7521) at a density of 150,000 cells per 6.5 mm Transwell (Costar No. 3470, 24 well) or 350,000 cells per 12 mm Transwell (Costar No. 3460, 12 well) in E-ALI medium (44). Cells cultured on 24-well Transwells were used for all experiments except for RNA-seq analysis, for which we used 12-well Transwells to increase RNA yield. E-ALI medium was based on previous formulations with modifications to glucose (150 mg/dL; 8.3 mM), CaCl_2_ (1 mM), heparin (2 µg/mL), l-glutamine (2.5 mM), hydrocortisone (960 mg/mL), bovine pituitary extract (20 µg/mL), and Mg^2+^ (0.5 µM). E-ALI medium is changed every other day with washing of the apical surface. Using this protocol, monolayers were fully differentiated 14 days after transitioning to ALI.

Differentiated cells were infected at multiplicity of infection (MOI) 0.1 for 6 h with SARS-CoV-2 USA-WA1/2020 (BEI Resources, No. NR-52281), consistent with previously used conditions that avoid gross disruption of tight junctions and other cell structures that occur at MOI of 1 or higher (38, 39, 48). The cells were then washed with ALI medium and further incubated for 72 h. At 6, 24, 48, and 72 h postinfection, apical surfaces were washed with 0.2 mL of E-ALI and 0.5 mL of basal medium was collected, banked at −20°C for further analysis and the cells were re-fed. Virus production by infected cells was determined by analysis of plaque-forming units (PFUs) present in cell culture medium. In brief, at 24, 48, and 72 h post infection, the apical surface was washed with 150 μL medium/well, and basolateral medium was also collected. To measure PFUs, a dilution series of the media was produced and plated onto confluent Vero E6 cells (100 μL/well) that were incubated for 72 h and then stained with crystal violet. Data are presented as total PFUs secreted over the entire 72-h period. In all cases, virus shedding was only detected in apical washes, there was no virus present in basolateral media. In some experiments, virus infection was confirmed using a LAMP assay kit (New England BioLabs, No. E2019S) according to the manufacturer’s instructions.

### RNA-Seq Analysis

Differentiated noninfected AUD and non-AUD cells harvested from Transwells were flash-frozen and submitted to Azenta Life Sciences for RNA extraction and bulk RNA-Seq. Sequencing Configuration: library preparation, Illumina, 2×150 bp, ∼350M raw paired-end reads (∼105 GB), single index, per lane. RNA-seq reads were analyzed for differential gene expression, alternative splicing, and gene ontology (GO) enrichment analysis. The heat map of differentially expressed genes taking into account the false discovery rate (*P* adj. value <0.05) was generated using Galaxy Project. All samples had a similar distribution of normalized read counts (Supplemental Fig. S1). The complete set of gene expression analysis and detailed GO analysis is available in the Supplemental Material (see https://doi.org/10.6084/m9.figshare.23727489).

### Transepithelial Resistance and Dye Flux

Transepithelial resistance (TER) was measured using an EVOM Voltohmmeter (World Precision Instruments, No. EVOM2). Before measuring, cells were washed with Dulbecco’s phosphate buffered saline containing Ca^+2^ and Mg^+2^ (DPBS, Corning No. 21-030-CV) followed by a 15-min incubation at 37°C in Ringer’s solution (140 mM NaCl, 5 mM KCl, 0.36 mM K_2_HPO_4_, 0.44 mM KH_2_PO_4_, 1.3 mM CaCl_2_·2H_2_O, 0.5 mM MgCl_2_·6H_2_O, 4.2 mM NaHCO_3_, 10 mM Na HEPES, 10 mM glucose). To facilitate comparison between different cells, TER values were normalized to preinfection values obtained at *t* = 0 h for each condition examined. Dye flux measurements were performed using calcein and 10 kDa Texas Red dextran and quantified as previously described (49).

### Immunofluorescence Microscopy

Cells on Transwell permeable supports were rinsed with DPBS then fixed with 4% paraformaldehyde (PFA) for 10 min at RT in the dark. This was followed by a DPBS rinse and 2 min of fixation in 1:1 methanol/acetone at RT. Cells were then washed three times with DPBS. Cells were permeabilized in 0.5% Triton X-100/DPBS++ for 5 min, followed by two 5-min incubations in blocking solution (0.5% Triton X-100 and 5% Goat serum in DPBS). Primary antibodies were added (diluted in 3% BSA in DPBS) and incubated overnight at 4°C. The cells were washed three times with DPBS and then incubated for 1 h at RT with fluorescent secondary antibodies diluted in 3% BSA. The secondary antibodies were removed and the cells were further incubated for 10 min in Hoechst 33342 (Thermo Fisher No. 62249) diluted 1:1,000 in DPBS to stain nuclei. Cells were washed three times with DPBS, and Transwells were mounted on slides using Vectashield mounting solution (Vector Labs No. H-1000-10).

Primary antibodies used for immunofluorescence were as follows: mouse anti-zonula occludens-1 (ZO-1, Invitrogen No. 339100, 1:500 dilution), rabbit anti-β-catenin (Abcam ab32572, 1:400 dilution), Rabbit anti-claudin 7 (Abcam No. ab27487, 1:200 dilution). Secondary antibodies used for immunofluorescence were as follows: Alexa fluor 568 goat anti-mouse (Invitrogen A11031, 1:1,500 dilution), Alexa fluor 488 goat anti-rabbit (Invitrogen No. A11034, 1:1,500 dilution), Alexa fluor 488 anti-mouse (Invitrogen No. A11029, 1:1,500 dilution), Alexa fluor 568 anti-rabbit (Invitrogen No. A11036, 1:1,500 dilution). Images were taken using either a Nikon Ti Eclipse with epifluorescence and processed using three-dimensional (3-D) deconvolution or a Nikon A1R confocal and processed using Nikon Elements and Image J. Fluorescence intensity of immunostained samples was measured for three fields each from three biological replicates and normalized to values obtained for uninfected, non-AUD samples.

For cell area quantitation, confocal images were processed in Image J using a macro to isolate the ZO-1 channel and create max Z projections. The max Z projections were uploaded in Cell Profiler, and a pipeline was created to threshold, dilate, and invert the images. Cells were then identified and cell area was calculated.

### Cytokine Analysis

Apical washes and basolateral medium that were collected at 6, 24, 48, and 72 h after SARS-CoV-2 infection were analyzed and the total amount of cytokine secreted was determined by measuring cytokine concentration multiplied by total volume. E-ALI medium collected at each timepoint following SARS-CoV-2 infection was analyzed using the MILLIPLEX Human Cytokine/Chemokine/Growth Factor Panel A kit (Cat. No. HCYTA-60K-PX38) according to the manufacturer’s instructions, modified to accommodate the BSL-3 facility. After streptavidin-phycoerythrin incubation, all wells on the assay plate were washed twice with assay buffer followed by the addition of 200 μL of 4% PFA and the samples were further incubated for 17 h at 4°C to denature any virus that was present before analysis. Data represent the total pg secreted at different timepoints and are also presented as the total pg secreted apically or basolaterally over a 72-h period.

### Statistics

Statistics were calculated using GraphPad Prism 8.0 and significance was determined using one-way ANOVA with using one-way ANOVA with Tukey multiple comparisons test or with Fisher’s least significant difference (LSD) test, as indicated in figure legends.

## RESULTS

### AUD and Non-AUD Airway Cells Have Differential Gene Expression Profiles

In this study, we used cells derived from airway brushings obtained from four individuals with AUD and three non-AUD individuals. The best-matched samples available consisted of lung brushings from Black or African American males and there was one AUD sample from a female donor (Table 1). Smoking status varied for each subject but in aggregate, these samples had comparable representative nonsmokers and smokers. These airway epithelial cells were proliferated as basal cells and then differentiated using ALI cultures as described in methods.

**Table 1.** Sample donor characteristics

Sample ID	Age	Sex	Height, in.	Weight, lbs	BMI	Race	Ethnicity	Current Smoker?	Pack Year History
*AUD 1*	50	Male	65	145	24.1	Black or African American	Not Hispanic or Latino	Yes	3
*AUD 2*	53	Male	71	201	28.0	Black or African American	Not Hispanic or Latino	Yes	26
*AUD 3*	51	Male	71	173	24.1	Black or African American	Not Hispanic or Latino	No	0
*AUD 4*	43	Female	69	263	38.8	Black or African American	Not Hispanic or Latino	No	0
*Non-AUD 1*	20	Male	77	195	23.1	Black or African American	n/a	No	0
*Non-AUD 2*	29	Male	66	172	27.8	Black or African American	Not Hispanic or Latino	Yes	4
*Non-AUD 3*	58	Male	66	135	21.8	Black or African American	Not Hispanic or Latino	Yes	45

RNAseq, barrier function and cytokine analysis were done using cells from donors *AUD 1*, *AUD 2*, *AUD 3*, *non-AUD 1*, *non-AUD 2*, and *non-AUD 3*. Immunofluorescence analysis was done using cells from donors *AUD 1*, *AUD 2*, *AUD 4*, *non-AUD 1*, *non-AUD 2*, and *non-AUD 3*. AUD, alcohol use disorder; BMI, body mass index.

**Figure 1.**
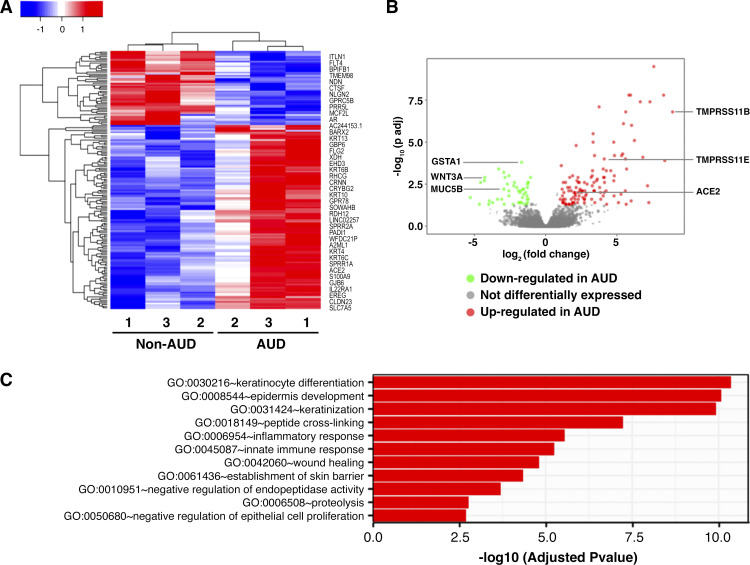
Differentiated bronchial cells from patients with non-alcohol use disorder (AUD) and AUD were subject to RNA sequencing. *A*: heat map representation of all 164 differentially expressed genes. Downregulated genes are in blue and upregulated genes are in red, in respect to AUD cells. Select differentially expressed (DE) genes are located on right. *B*: volcano plot representation of all genes. Green dots represent downregulated genes in AUD cells, red dots represent upregulated genes in AUD cells, and gray dots represent genes that were not differentially expressed. Severe acute respiratory syndrome coronavirus 2 (SARS-CoV-2) receptor genes were upregulated in AUD cells. Genes required for proper function and maintenance of bronchial cells were downregulated in AUD cells. *C*: differentially expressed genes were clustered by their gene ontology. Enrichment of gene ontology terms was tested using Fisher exact test (GeneSCF v1.1-p2). Shown are top 11 significantly enriched gene ontology terms with an adjusted *P* value less than 0.05 in the differentially expressed gene set to account for false discovery rate. ACE, angiotensin-converting enzyme.

To determine whether there were AUD-dependent differences in gene expression, we analyzed cultures derived from *AUD 1*–*3* and *non-AUD 1*–*3* by RNA-seq. Normalized read counts were comparable for all six samples (Supplemental Fig. S1). As shown by hierarchical clustering (Fig. 1*A*), the samples stratified by AUD status showed distinct patterns of upregulated and downregulated genes. Further analysis by volcano plot showed that there were 117 upregulated genes and 47 downregulated genes in AUD cells compared with non-AUD cells (Fig. 1*B*). A full list of differentially expressed (DE) genes can be found in Supplemental Table S1.

Shown in Fig. 1*C* is gene ontology (GO) enrichment analysis reflecting the top 11 GO terms, of which five relate to epidermal differentiation and barrier function and two relate to inflammation. The DE genes categorized into these GO categories can be found in Tables 2 and 3. Among the epidermally related upregulated genes are small proline-rich proteins (SPRRs) that are largely expressed in the epidermis and have bactericidal properties (50). Moreover, SPRR3 has been found to play a role in allergic airway inflammation, where knockdown of SPRR3 reduced the number of inflammatory cells in the bronchoalveolar lavage (BAL) fluid (50).

**Table 2.** Genes upregulated by AUD related to epidermal differentiation

Gene Name	Log 2 Fold Change	Adj. *P* Value
ABCA12	2.09	0.02
CALML5	4.57	0
CRNN	7.32	0
EMP1	2.29	0.03
EREG	2.2	0
FLG2	5.11	0.04
GRHL3	2.27	0
KLK6	3.85	0.01
KLK7	3.75	0
KRT10	1.66	0.05
KRT13	3.87	0.03
KRT16	3.23	0.02
KRT24	8.36	0
KRT4	3.63	0
KRT6B	3.79	0
KRT6C	3.27	0
KRT78	6.64	0
SCEL	2.42	0.01
SPINK5	4.83	0
SPINK7	5.66	0.05
SPRR1A	6.23	0
SPRR1B	4.06	0
SPRR2A	5.96	0
SPRR2D	5.62	0
SPRR2E	5.68	0
SPRR3	7.59	0
TGM1	2.73	0.02

Alphabetically sorted list of genes enriched in alcohol use disorder (AUD) cells, as determined by gene ontology (GO) analysis for GO:0030216: keratinocyte differentiation, GO:0008544: epidermis development, GO:0031424: keratinization, GO:0018149: peptide cross-linking, GO:0061436: establishment of skin barrier.

**Table 3.** Genes upregulated by AUD related to inflammation

Gene Name	Log 2 Fold Change	Adj. *P* Value
BDKRB2	1.24	0
C6	2.75	0.01
CLEC7A	1.28	0.01
ECM1	3.57	0
IL1RN	2.07	0.01
IL22RA1	2.72	0
IL36A	6.82	0
IL36RN	4.04	0.01
KRT16	3.23	0.02
MGLL	2.29	0
PGLYRP4	1.22	0.04
S100A12	4.13	0
S100A8	4.78	0
S100A9	2.28	0.01

Alphabetically sorted list of genes enriched in alcohol use disorder (AUD) cells, as determined by gene ontology (GO) analysis for GO:0006954: inflammatory response and GO:0045087: innate immune response.

The genes upregulated by AUD related to inflammation (Table 3) reflect a relatively mild phenotype and include receptor antagonist genes such as IL1RN and IL36RN. Genes classically associated with inflammation, such as TNFα, IL-1β, IFNγ, and IL-6, were comparably expressed by AUD and non-AUD cells at baseline. However, there were some proinflammatory genes that were upregulated, most notably S100A8, S100A9, and S100A12. These genes are damage-associated molecular pattern (DAMP) molecules (51) that have been linked to several pathological states, including severe asthma (52, 53).

Among the genes upregulated in AUD are several that have been implicated in SARS-CoV-2 infection, including angiotensin-converting enzyme (ACE)2, which encodes the receptor that binds the virus spike protein, and two TMPRSS isoforms: TMPRSS11B and TMPRSS11E. Although the TMPRSS2 isoform is best known for activating the SARS-CoV-2 spike protein, one study found enhanced fusion between 293 cells expressing SARS-CoV-2 S protein and 293 cells expressing hACE2 and TMPRSS11E (54). Taken together, these data suggested that AUD status of donor cells is an independent determinant of gene expression of bronchial epithelial cells that may impact their response to SARS-CoV-2 infection.

### SARS-CoV-2 Infection Impairs Barrier Function of AUD Cells

**Figure 2.**
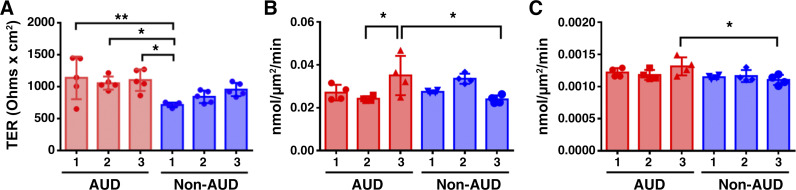
Baseline barrier function of alcohol use disorder (AUD) and non-AUD airway cells. *A*: transepithelial resistance (TER) values of all samples immediately prior to infection. *Non-AUD 1* had a lower TER compared with *AUD 1* (**P = 0.007) and *AUD 2* (*P = 0.04) and *AUD 3* (*P = 0.01). *n* = 3 biological replicates per condition consisting of five Transwells per group. Values represent means ± SD in each case. *B* and *C*: cells were incubated with a mixture of calcein (*B*) and 10 kDa Texas Red Dextran (*C*) over a 2-h period and the rate of flux from the apical to basolateral surface was determined. *B*: the amount of calcein flux was higher for *AUD 3* vs. *AUD 2* (*P = 0.02) and vs. *non-AUD 3* (*P = 0.02). *C*: the amount of 10 kDa Texas Red Dextran flux was higher for *AUD 3* than *non-AUD 3* (*P = 0.03). *n* = 3 biological replicates per condition consisting of four Transwells per group. Samples were analyzed using one-way ANOVA with Tukey multiple comparisons test.

Prior to infection, we measured transepithelial resistance (TER) and paracellular flux of calcein and 10 kDa Texas Red dextran as indices of barrier integrity. By and large, barrier function was comparable when comparing bronchial epithelial cells derived from AUD and non-AUD brushings, with some individual variability (Fig. 2). This is in contrast to alveolar epithelial cells, which consistently have impaired barrier function when chronically exposed to alcohol in vivo (30).

**Figure 3.**
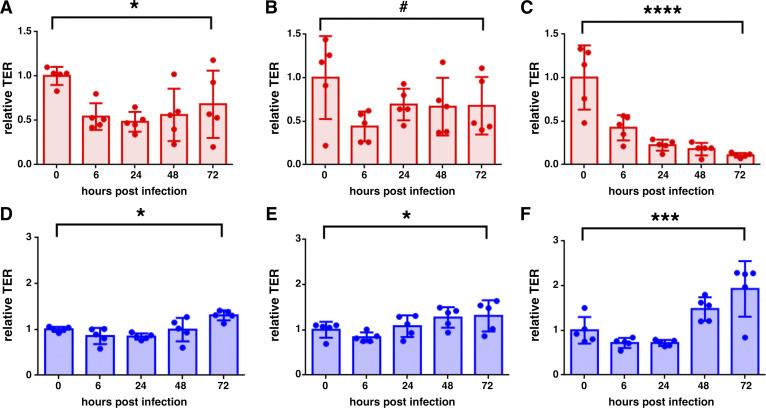
Alcohol use disorder (AUD) cells have impaired barrier function following severe acute respiratory syndrome coronavirus 2 (SARS-CoV-2) infection. Differentiated bronchial cells from subjects with AUD (*A*–*C*, red) and non-AUD (*D*–*F*, blue) were infected with SARS-CoV-2. Transepithelial resistance (TER) was measured immediately prior to infection (*t* = 0) and at 6, 24, 48, and 72 h postinfection, normalized to values at *t* = 0. AUD cells showed decreased barrier function 72 h after infection (*A*, *P = 0.04; *B*, #*P* = 0.11; *C*, ****P < 0.0001). By contrast, non-AUD cells showed increased barrier function 72 h after infection (*D*, *P = 0.034; *E*, *P = 0.046; *F*, ***P = 0.0002). *n* = 3 biological replicates consisting of five Transwells per group. Values represent means ± SD in each case. Samples were analyzed using one-way ANOVA with Fisher’s least significant difference (LSD) test.

The cells with TER values shown in Fig. 2*A* were infected with the Washington strain of SARS-CoV-2 at 0.1 MOI. TER values were measured at intervals over the course of a 72-h time course and normalized to baseline values to facilitate comparisons for each condition examined (Fig. 3). In each of the three different AUD isolates, SARS-CoV-2 infection decreased TER (Fig. 3, *A–C*). On the other hand, the three different non-AUD cell isolates showed an increase in TER 72 h following infection (Fig. 3, *D–F*).

**Figure 4.**
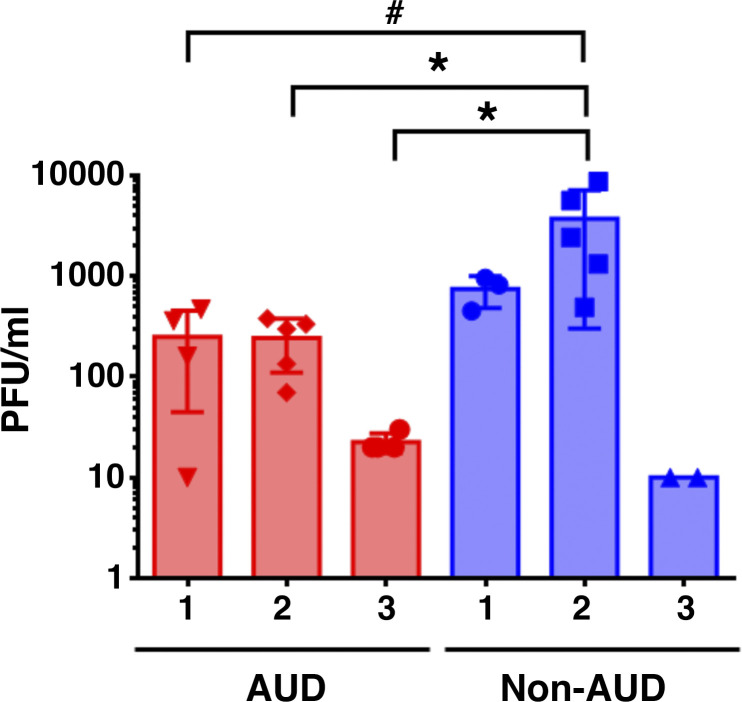
Virus shedding from infected cells. Alcohol use disorder (AUD) and non-AUD cells were infected with severe acute respiratory syndrome coronavirus 2 (SARS-CoV-2) at multiplicity of infection (MOI) 0.1 for 6 h. Medium was collected from apical surface washes over a 72-h period and total plaque forming units (PFU) were determined using Vero E6 cells as described in methods. *Non-AUD 2* cells showed the highest levels of virus shedding, which was higher than the amount of virus shed from AUD cells (*P = 0.04; #*P* = 0.06) *n* = 3 biological replicates consisting of 2–5 Transwells per group. Samples were analyzed using one-way ANOVA with Tukey multiple comparisons test. Basolateral medium did not contain detectable levels of SARS-CoV-2.

Differences in TER postinfection did not correlate with the level of virus production, since infected AUD cells produced comparable or lower levels of SARS-CoV-2 as compared with infected non-AUD cells (Fig. 4). Note that virus secretion was only detected from the apical surface; there was not any virus detectable in the basolateral medium. The lack of detectable basolateral virus was consistent with dye flux measurements from infected cells, which did not show enhanced paracellular substrate diffusion in response to infection (Supplemental Fig. S2), in contrast to TER measurements (Fig. 3).

**Figure 5.**
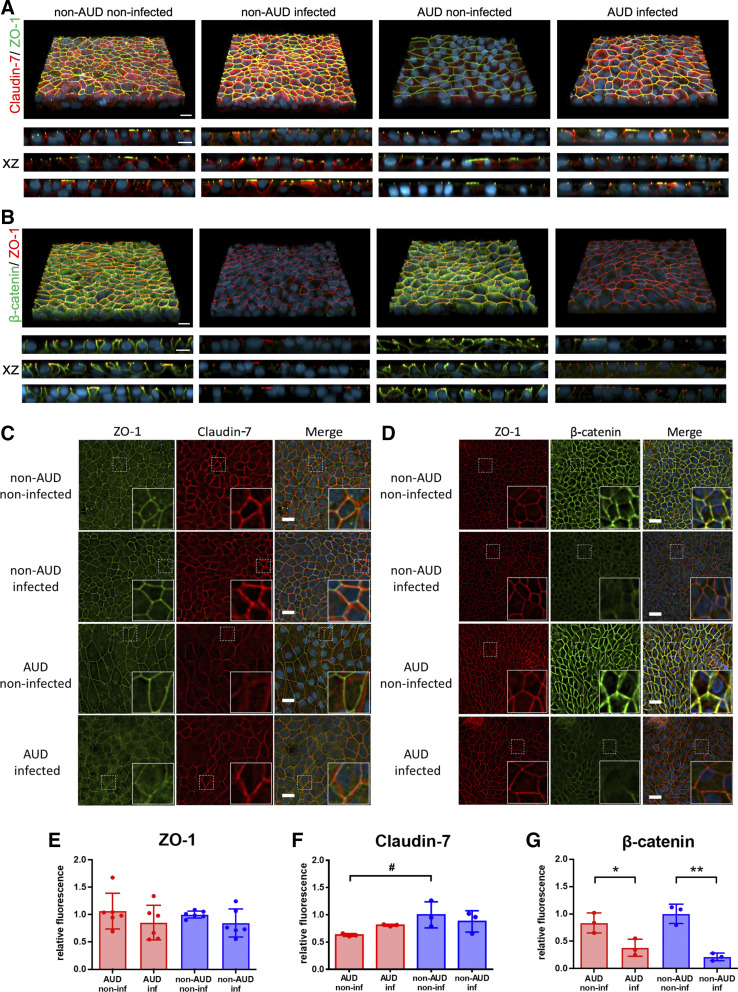
Changes to non-alcohol use disorder (AUD) and AUD bronchial cell junctions in response to severe acute respiratory syndrome coronavirus 2 (SARS-CoV-2) infection. *A*–*D*: cells 72 h postinfection or mock infection were fixed and immunostained for zonula occludens-1 (ZO-1, green) and claudin-7 (red) and DAPI (blue) (*A* and *C*) or ZO-1 (red), β-catenin (green), and DAPI (blue) (*B* and *D*) and imaged by confocal (*A* and *B*) and deconvolution (*C* and *D*) fluorescence microscopy. *A* and *B*: top panels show a three-dimensional (3-D) projection, the *bottom* panels represent *xz* projections from three biological replicates for each condition. Bar, 10 μm. *C* and *D*: *xy* projections of representative images. White dotted squares represent the location of insets in the bottom right corner of each image. Bar, 20 micron. *E*–*G*: SARS-CoV-2 infection had little effect on relative fluorescence intensity of ZO-1 (*E*) and claudin-7 (*F*), however there was a significant decrease in total β-catenin (*G*) 72 h postinfection (*P = 0.027, **P = 0.001, *n* = 3 biological replicates consisting of 3 image fields each). Also, there was a trend towards less claudin-7 in noninfected AUD cells compared with noninfected non-AUD cells (#*P* = 0.076, *n* = 3 biological replicates consisting of 3 image fields each), although this difference diminished 72 h postinfection. Values represent means ± SD in each case. Samples were analyzed using one-way ANOVA with Tukey multiple comparisons test.

Taken together, these data support a model where the AUD bronchial epithelial cell junctions are more sensitive to SARS-CoV-2 infection than non-AUD cell junctions. To determine whether the differential effects of SARS-CoV-2 infection on TER were due to differences between AUD and non-AUD cells in epithelial junction organization, we used confocal and deconvolution immunofluorescence microscopy (Fig. 5). Over the course of our experiments, the tight junction scaffold protein zonula occludens-1 (ZO-1) remained predominantly tight junction-associated. Also, total levels of ZO-1 were unchanged by SARS-CoV-2 infection (Fig. 5*E*).

We also examined one of the major transmembrane tight junction proteins responsible for bronchial epithelial barrier function, claudin-7 (55). At baseline, there was a trend toward more claudin-7 present in non-AUD than in AUD bronchial epithelial cells (Fig. 5*F*). Note that most of the claudin-7 expressed by bronchial cells is present on the lateral plasma membrane (Fig. 5*A*, *xz*), that is not colocalized with ZO-1 and does not contribute to barrier function (56). Having less claudin-7 associated with tight junctions in infected AUD cells (Fig. 5*C*) is anticipated to result in decreased barrier function. It appeared that there might be a difference in bronchial epithelial cell size, related to AUD status, however, quantitative analysis determined that this was not significant (Supplemental Fig. S3).

In contrast to ZO-1 and claudin-7, levels of β-catenin, an adherens junction scaffold protein, were significantly diminished in response to SARS-CoV-2 infection (Fig. 5, *B*, *D*, and *G*). A similar effect of SARS-CoV-2 on β-catenin expression has been reported for infected endothelial cells (57, 58) and has been implicated in the disruption of vascular barrier function due to COVID-19. However, infection had a similar effect on total β-catenin in AUD and non-AUD cells, suggesting that this does not account for the differential effect of infection on barrier function of bronchial epithelial cells. Instead, the decrease in airway epithelial β-catenin following SARS-CoV-2 infection more likely reflects another cell response that is unaffected by AUD, such as translocation of junction localized β-catenin to mediate wnt signaling (59).

### Differential Secretion of Cytokines by Infected AUD and Non-AUD Cells

**Figure 6.**
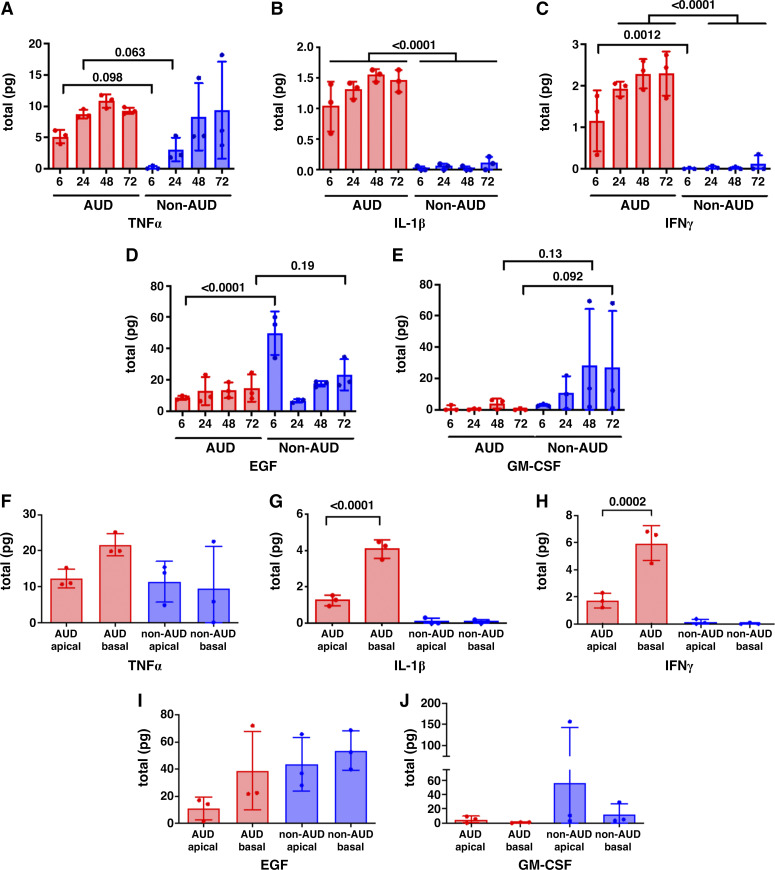
Proinflammatory cytokines were preferentially secreted by alcohol use disorder (AUD) cells in a polarized manner in response to infection. Differentiated bronchial cells from patients with non-AUD and AUD were infected with severe acute respiratory syndrome coronavirus 2 (SARS-CoV-2) and analyzed for cytokine secretion at 6, 24, 48, and 72 h postinfection (*A*–*E*) or analyzed for apical and basolateral secretion over the entire time course (*F*–*J*). There was a trending or significant increase in proinflammatory cytokine secretion (TNFα, IL-1β, and IFNγ) by AUD cells at some or all timepoints. There was a trending or significant increase in anti-inflammatory cytokines by non-AUD cells at some timepoints. AUD cells secreted significantly more basolateral IL-1β, and IFNγ. There was equivalent apical and basolateral secretion of TNFα, epidermal growth factor (EGF) and granulocyte macrophage-colony stimulating factor (GM-CSF) by AUD cells. For all five cytokines, non-AUD cells secreted comparable amounts apically and basolaterally. *n* = 3 biological replicates consisting of samples from three Transwells per group. Values represent means ± SD in each case. Samples were analyzed using one-way ANOVA with Fisher’s least significant difference (LSD) test.

We then performed multiplex analysis of cytokine secretion by SARS-CoV-2 infected cells. In general, most of the proinflammatory cytokines we measured showed higher levels of secretion by AUD cells during the initial 72-h period postinfection as compared with non-AUD cells (Fig. 6, Supplemental Fig. S4). Notable among these cytokines are TNFα, IL-1β, and IFNγ (Fig. 6, *A–C*), where differences in IL-1β and IFNγ secretion between AUD and non-AUD cells were significant and there was a trending higher level of TNFα secretion by AUD cells at 6 and 24 h after infection. These cytokines have been found to be upregulated in patients with COVID-19 (60) and have the capacity to cause barrier dysfunction (61, 62). This is consistent with the effect we observed on barrier function in Fig. 3. On the other hand, non-AUD cells secreted more epidermal growth factor (EGF) over the 72-h time course compared with AUD cells (Fig. 6*D*). In addition, there was a trend toward increased granulocyte macrophage-colony stimulating factor (GM-CSF) secretion by infected non-AUD cells compared with AUD cells (Fig. 6*E*). Both EGF (63, 64) and GM-CSF (21)) have been associated with improved lung epithelial barrier function, which is consistent with the effect of SARS-CoV-2 infection on non-AUD cells observed in Fig. 3.

An additional 16 cytokines were preferentially secreted by AUD cells at least at one timepoint (Supplemental Fig. S4). It is noteworthy that AUD cells secreted more IL-17A, IL-17F, and IL-25/IL-17E (Supplemental Fig. S4, *L*, *M*, and *P*), as IL-17 strongly correlates with severe COVID-19 (65). Nine additional cytokines were found to be equivalently secreted from AUD and non-AUD cells during the 72-h time course, including IL-1α and IL-13 (Supplemental Fig. S5). Total IL-6 secretion was also comparable for AUD and non-AUD cells over the 72-h period, however, there appeared to be a lag in IL-6 secretion by non-AUD cells (Supplemental Fig. S5*F*). Other cytokines equivalently secreted by AUD and non-AUD cells included IL-4 and TNFβ (Supplemental Fig. S5). AUD cells also secreted three anti-inflammatory cytokines in response to infection: IL-1RA, IL-10, and IL-22 (Supplemental Fig. S5). Of these, it is noteworthy that elevated IL-1RA and IL-10 have been associated with severe COVID-19, likely reflecting an attempt by the host to mount a protective response (66–68).

We also assessed apical and basolateral secretion of cytokines and found that AUD cells had significantly greater basolateral secretion of IFNγ and IL-1β, whereas secretion of TNFα, EGF, GM-CSF was not significantly polarized (Fig. 6, *F–J*). Seventeen additional cytokines from our multiplex analysis were found to be secreted in a polarized manner (Supplemental Fig. S6). We observed a variety of polarity profiles, including eight cytokines that were significantly secreted basolaterally from AUD cells whereas five cytokines were significantly secreted basolaterally from non-AUD cells. Chemokine (C-C motif) ligand 5 (CCL5) and macrophage colony-stimulating factor (M-CSF) were significantly secreted apically from AUD cells whereas IL-7 and IL-22 were significantly secreted basolaterally in both AUD and non-AUD cells (Supplemental Fig. S6, *B*, *F*, *M*, and *O*). On the other hand, eight cytokines secreted by infected cells did not show significant polarity in secretion (Supplemental Fig. S7).

Taken together, these results support a model in which AUD cells were primed for an early, enhanced innate immune response to SARS-CoV-2 infection which can have a deleterious effect on cell function as compared with non-AUD cells.

## DISCUSSION

The effect of SARS-COV-2 infection on human primary bronchial cells in culture has been examined by others (37–41, 43), however, this study is the first analysis of the effects of AUD on cell responses to SARS-CoV-2 infection. Our data support a model where AUD and non-AUD differ in their inflammatory response to SARS-CoV-2 infection. This was not due to AUD cells having a strong proinflammatory phenotype at baseline, but instead reflected a phenotypic shift that sensitized them to mount an earlier innate inflammatory response to SARS-CoV-2 infection than non-AUD cells.

Our study is the first to focus on samples obtained from Black or African American subjects concerning the effects of AUD on SARS-CoV-2 infection (Table 1). In the United States, African American populations have disproportionally higher rates of SARS-CoV-2 infection, hospitalization, and COVID-19-related mortality, according to a systematic review of numerous studies (69). This is a multifactorial issue due in part to adverse social determinants of health and increased prevalence of comorbidities (70). Although we were unable to adjust for smoking in our study, we did have two smokers represented in each of the AUD and non-AUD samples, making the percentage of smokers 66%. This percentage of smokers in the AUD patient samples is equivalent to the percentage of chronic alcohol users who smoke at least one pack of cigarettes a day, which is 70% (71).

RNA-seq analysis of our samples revealed 164 DE genes with the majority upregulated in AUD cells. Note that both AUD and non-AUD cells were cultured in the absence of added ethanol in the medium, indicating that any changes in gene expression were persistent during the cell culture and differentiation process, likely due to epigenetic reprogramming as a result of AUD. Gene ontology (GO) enrichment analysis showed that the most significant GO terms enriched for AUD samples are related to epidermal/keratinocyte differentiation and barrier function. In particular, there was enhanced expression of genes related to a cornified barrier, including CRNN, several SPRR genes and FLG2. This, in combination with decreased MUC5B expressed by AUD cells, suggests that the molecular nature of AUD and non-AUD cells is likely to be different, even though their barrier function was numerically equivalent. As one example of how this could have functional ramifications related to the impact of AUD on lung function, increased SPRR3 expression has previously been associated with lung inflammation (50). We also found that CEACAM5, a gene encoding a cell adhesion protein, was upregulated in AUD cells. CEACAM5 is also upregulated in bronchial cells from patients with severe type-2 asthma (72), further suggesting a proinflammatory phenotype.

In addition, AUD cells had decreased expression of GSTA1, WNT3A, and MUC5B, which are enhanced in non-AUD cells. Since these genes play roles in protection from oxidative stress, airway repair, and clearance of respiratory particulates and pathogens, respectively (73, 74), the decreased expression of these genes is consistent with AUD airway epithelial cells being sensitized to injury and/or infection.

Bailey et al. (75) performed RNA-seq analysis of unexpanded bronchial brushings from 19 non-AUD people and 18 people with AUD. We found that there were some DE genes in our RNAseq analysis that were represented in the study by Bailey et al. dataset (Table 4, uncorrected for smoking status); however, this required using a less stringent measure of significance for the Bailey dataset, namely DEs with a significant raw *P* value, even though the adj. *P* value was not significant, meaning they did not surpass the false discovery rate. One explanation for an overall difference in the DE datasets could be demographic differences between sample donors in the two different studies. Another difference is that we analyzed cells that were propagated and differentiated as opposed to brushings that were directly analyzed, so they could represent different classes of cell phenotypes.

**Table 4.** Genes altered by AUD status

	Current Study			Bailey et al. (75)		
Gene Name	Log 2 Fold Change	*P* Value	Adj. *P* Value	Log 2 Fold Change	*P* Value	Adj. *P* Value
CEACAM7	4.82	0	0	2.58	0.014	0.180
S100A8	4.78	0	0	2.10	0.021	0.210
CYSRT1	3.43	0	0	1.06	0.018	0.195
CEACAM5	3.39	0	0.01	4.18	0.001	0.055
PADI1	3.33	0	0	1.55	0.015	0.186
ABCA12	2.09	0	0.02	2.34	0.010	0.160
CRYBG2	1.62	0	0.01	1.32	0.017	0.192
PLBD1	1.41	0	0.05	0.77	0.008	0.142
BDKRB2	1.24	0	0	0.42	0.052	0.309
GPRC5B	−1.63	0	0.02	−0.84	0.023	0.223
MUC5B	−2.93	0	0.01	−2.05	0.004	0.102
CYP2A13	−3.99	0	0.05	−2.09	0.002	0.085
ITLN1	−4.57	0	0	−3.18	0.011	0.161

Examples of genes comparably differentially regulated in our comparison of cultured alcohol use disorder (AUD) and non-AUD cells and data from Bailey et al. (75), which were processed for AUD status, but not smoking status. Although both sets of data had significant *P* values, the adjusted *P* value for these genes was not significant for the Bailey et al. data, which is a limitation of this analysis.

After infection with SARS-CoV-2, cells from patients with AUD had a robust inflammatory response, including higher levels of TNFα, IL-1β, and IFNγ secreted than infected non-AUD cells. Several other proinflammatory cytokines were also preferentially secreted by infected AUD cells as compared with non-AUD cells (Supplemental Fig. S4). However, there were also several cytokines that were comparably secreted by infected AUD and non-AUD cells. For instance, over the 72-h period following infection, IL-6 secretion was comparable for AUD and non-AUD cells (Supplemental Fig. S5*F*), although AUD cells had an earlier IL-6 response than non-AUD cells. Other studies examining SARS-CoV-2-exposed bronchial cells have also showed that IL-6 was produced at 72 h after infection (37, 40, 43), consistent with the response we observed in non-AUD cells. Although there is a correlation of systemic IL-6 levels with disease severity (66, 76), it is not the sole determinant and an overall inflammatory profile should be considered.

Surprisingly, we found that SARS-CoV-2 infected, non-AUD cells secreted two protective cytokines, EGF and GM-CSF, that were less prominent in infected AUD cells. EGF in particular has been shown to promote lung epithelial cell barrier function (63, 64). Moreover, administration of EGF to septic mice has been shown to lessen the severity of sepsis, even in alcohol-fed mice, in part by protecting gut barrier function (77).

Interpreting roles for GM-CSF in bronchial epithelial cell behavior is complex, since it has both pro- and anti-inflammatory effects, depending on the amount, context, and presence of other inflammatory mediators (78). Consistent with the protective effect of GM-CSF, GM-CSF-deficient mice exhibit pulmonary alveolar proteinosis (PAP) (79), which ultimately led to therapies including inhaled GM-CSF to treat this disease (80). There is also evidence that ARDS survival correlates with the amount of GM-CSF present in lung lavage fluid (21, 81). This is due to stimulation of the PU.1 transcription factor by autocrine stimulation of lung epithelial cells by GM-CSF and this pathway was found to be impaired as a result of chronic alcohol ingestion in a rodent model (82). This would be consistent with impairment of GM-CSF signaling by AUD cells as a contributor to barrier dysfunction due to SARS-CoV-2 and supports the potential for GM-CSF administration as a therapeutic approach in severe COVID-19 (83, 84). However, the use of administered GM-CSF to treat non-COVID ARDS has had mixed success, where it was shown to improve the ratio of arterial oxygen partial pressure to fractional inspired oxygen ($\text{P}{\text{a}}_{{\text{O}}_{2}}$/$\text{F}{\text{I}}_{{\text{O}}_{2}}$) (85), but it did not increase the number of ventilator-free days in patients with ARDS (86).

We found that AUD cells showed a significant decrease in TER in response to SARS-CoV-2 infection, however non-AUD cells showed an increase in TER. Previous analysis of infected airway cells has shown a minimal effect of SARS-CoV-2 infection on TER over a 6-day period (39) or over a 30-day period following an initial drop, with some fluctuations (38). Although the enhancement of TER by non-AUD cells was unexpected, it was consistent with another study demonstrating that SARS-CoV-2 infection of bronchial epithelial cells showed a transient decrease in TER followed by a rebound to higher TER when measured over a 7-day time course postinfection (48).

Another likely mechanism of diminished barrier function is influence of viral proteins on assembly of the tight junction complex, even in the absence of viral shedding. In particular, interference of the SARS-CoV envelope protein (E protein) with ZO-1 association with tight junctions was originally demonstrated for SARS-CoV-1 (87). Subsequently, SARS-CoV-2 E protein has been found to bind to ZO-1 as well (88–91). Whether this is occurring in SARS-CoV-2 infected airway cells to interfere with tight junctions remains to be determined. Disruption of ZO-1 can also influence the distribution of claudin-7 between the tight junction (barrier forming) and lateral (nonbarrier forming) pools. Regardless of the mechanism, cell polarity is largely retained, since infected airway cells in vitro show low levels of basolateral virus shedding relative to apical shedding (38, 48) and cytokine secretion is also polarized, as seen here and in other reports (37, 40). Consistent with this, we did not see an effect of SARS-CoV-2 infection on dye flux, suggesting that changes in TER were related to changes in tight junction composition as opposed to disassembly and cell depolarization.

In contrast to ZO-1 and claudin-7, levels of β-catenin, an adherens junction scaffold protein, were significantly diminished in response to SARS-CoV-2 infection irrespective of donor sex and whether the cells were from people with or without AUD (Fig. 5, *B*, *D*, and *G*). A similar effect of SARS-CoV-2 on β-catenin expression has been reported for infected endothelial cells (57, 58) and has been implicated in the disruption of vascular barrier function due to COVID-19. However, since SARS-CoV-2 infection had a similar effect on total β-catenin in AUD and non-AUD cells, this does not account for the differential effect of infection on barrier function of bronchial epithelial cells. Instead, the decrease in airway epithelial β-catenin following SARS-CoV-2 infection more likely reflects another cell response. For instance, decreased expression and junction localization of β-catenin could alter airway cell responses to infection by having an impact on wnt signaling (59).

We found that the effects of AUD on human airway epithelial cell responses to SARS-CoV-2 infection were maintained by cultured cells, and did not require the presence of alcohol in the culture medium. This finding is consistent with our data using lung epithelial cells isolated from alcohol-fed rodents (30, 31, 33) and suggests that the cells may be epigenetically reprogrammed in response to chronic alcohol exposure. In fact, alcohol consumption has been linked to epigenetic modification of the central nervous system as a mechanism underlying addiction (92), which further suggests that epigenetic reprogramming also can occur in the lung in response to AUD. Consistent with this possibility, it has previously been shown that alcohol inhibits Thy-1 expression by lung fibroblasts by DNA methylation induced by TGF-β1 (93, 94). The effects of epigenetic reprogramming of lung epithelia by alcohol remain to be determined.

Our findings that non-AUD cells had a relatively mild response to SARS-CoV-2 infection are likely to reflect the cell culture conditions we used. Here, we used medium that supports bronchial cell differentiation and also contains normal resting glucose levels (44). However, several media commonly used to support airway epithelial cell differentiation have high glucose concentrations, including media based on LHC Basal:DMEM-H and Pneumocult-ALI both of which contain ∼300 mg/dL of glucose (44). Thus, one consideration in interpreting results obtained with cultured airway epithelial cells is that their response to SARS-CoV-2 infection may be sensitive to medium glucose content, which would be consistent with diabetes as a risk factor for increased severity of COVID-19 (65).

Here, we compared primary bronchial epithelial cells derived from patients with AUD and non-AUD that were grown, differentiated, and treated under the same conditions to demonstrate that AUD cells showed early sensitivity to SARS-CoV-2 infection. As early onset of severe disease is a likely determinant of further disease progression, our data add AUD as a risk factor for increased severity of COVID-19-related illness (15) due to the combined impact of alcohol and SARS-CoV-2 infection on airway epithelial barrier function and inflammation. This underscores the importance of considering AUD status when treating patients with COVID-19 and the likely utility of targeting the lung epithelium when considering treatment options.

## DATA AVAILABILITY

Data will be made available upon reasonable request.

## SUPPLEMENTAL DATA

10.6084/m9.figshare.23729376 Supplemental Figs. S1–S7: https://doi.org/10.6084/m9.figshare.23729376.

10.6084/m9.figshare.23729376 Supplemental Table S1: https://doi.org/10.6084/m9.figshare.23729376.

10.6084/m9.figshare.23727489 Supplemental Datafiles: https://doi.org/10.6084/m9.figshare.23727489.

## GRANTS

This project was supported by NIH R01-AA025854 (to M.K. and C.A.E.), R01-HL158979 (to M.K.), F31-AA029000 (to K.F.E.), K08 AA024512 (to B.S.S.), U.S. Department of Veterans Affairs (VA) 1IK2CX000643 (to A.J.M.), and the Indo-U.S. Science & Technology Forum (IUSSTF) Virtual Networks for COVID-19 (Ref.: IUSSTF/VN-COVID/107/2020), India (to M.K. and J.D.S.).

## DISCLOSURES

No conflicts of interest, financial or otherwise, are declared by the authors.

## AUTHOR CONTRIBUTIONS

K.F.E., R.C.E., A.J.M., C.A.J., J.M.L., S.M.T., and M.K., conceived and designed research; K.F.E., R.C.E., M.E.J.L., R.C.R., A.J.M., E.K.L., I.K.C., S.K.J., C.A.J., A.-G.B.-B., S.M.T., C.A.E., and M.K. performed experiments; K.F.E., R.C.E., M.E.J.L., R.C.R., E.K.L., I.K.C., S.K.J., C.A.J., A.-G.B.-B., J.M.L., S.M.T., C.A.E., M.K., analyzed data; K.F.E., R.C.E., R.C.R., J.D.S., B.S.S., I.K.C., S.K.J., C.A.J., A.-G.B.-B., J.M.L., S.M.T., C.A.E., and M.K. interpreted results of experiments; K.F.E., J.M.L., and M.K. prepared figures; K.F.E., R.C.E., A.-G.B.-B., J.M.L., C.A.E., and M.K. drafted manuscript; K.F.E., R.C.E., J.D.S., B.S.S., S.K.J., C.A.J., A.-G.B.-B., J.M.L., S.M.T., C.A.E., and M.K., edited and revised manuscript; K.F.E., R.C.E., M.E.J.L., R.C.R., J.D.S., A.J.M., B.S.S., I.K.C., S.K.J., C.A.J., A.-G.B.-B., J.M.L., S.M.T., C.A.E., and M.K. approved final version of manuscript.
